# Prediction of lymph node metastasis in oral tongue squamous cell carcinoma using the neutrophil‐to‐lymphocyte ratio and platelet‐to‐neutrophil ratio

**DOI:** 10.1002/jcla.23684

**Published:** 2021-05-04

**Authors:** Bo Wang, Junwen Liu, Zhengrong Zhong

**Affiliations:** ^1^ Department of Clinical Laboratory Shanghai Ninth People's Hospital Shanghai JiaoTong University School of Medicine Shanghai China; ^2^ Department of Clinical Laboratorial The Children's Hospital of Fudan University Shanghai China

**Keywords:** neutrophil‐to‐lymphocyte ratio (NLR), oral tongue squamous cell carcinoma (OTSCC), platelet‐to‐neutrophil ratio (PNR)

## Abstract

**Background:**

Lymph node metastasis in a variety of tumors is associated with systemic inflammatory markers. However, this association has not been reported in oral tongue squamous cell carcinoma (OTSCC). This study aimed to investigate how the preoperative neutrophil‐to‐lymphocyte ratio (NLR) and platelet‐to‐neutrophil ratio (PNR) in OTSCC patients correlated with the occurrence of OTSCC and lymph node metastasis.

**Methods:**

The data of 73 patients with primary OTSCC who underwent surgical resection were retrospectively analyzed. Patients with other malignant tumors, patients who had received radiotherapy or chemotherapy before surgery, and patients with active inflammation were excluded. The enrolled patients were divided into groups N_0_ (no early‐stage lymph node metastasis) and N_1_ (early‐stage lymph node metastasis). Venous blood samples were collected before surgery and at the third week after surgery and subjected to complete blood counting in a blood analyzer. Eighty‐seven healthy people were included as a control group. In addition, the NLR and PNR in OTSCC patients were compared with those in the controls, and the postoperative NLR and PNR of group N_0_ were compared with those of group N_1_.

**Results:**

The NLR was significantly higher in the OTSCC patients than the controls (*p* < 0.05). The area under the receiver operating characteristic curve was 0.595. Further comparison of the NLR and PLR between group N_0_ and group N_1_ showed that when NLR was ≤1.622, and the probability of early‐stage lymph node metastasis in OTSCC patients was 73.3%, and when PNR was >60.889, the probability was 86.7%. In re‐examination 3 weeks postoperatively, the NLR and PNR were not significantly different between groups.

**Conclusion:**

The NLR has certain reference value for the diagnosis of OTSCC. The preoperative NLR and PNR can be used to predict early‐stage lymph node metastasis in patients with histopathologically confirmed OTSCC.

## Graphical abstract

My study showed the neutrophil‐to‐lymphocyte ratio (NLR) and platelet‐to‐neutrophil ratio (PNR) could provide a reference for the development of OTSCC and OTSCC patients with lower NLR and higher PNR often accompanied by lymph node metastasis.

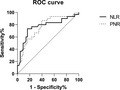

## 1 INTRODUCTION

Oral tongue squamous cell carcinoma (OTSCC) is one of the most common oral malignancies, and its incidence has been rising in recent years. Because the tongue is rich in blood vessels and lymphatic vessels, it is prone to lymph node metastasis. Therefore, the prognosis of OTSCC is poor, and the 5‐year survival rate is low.^1^, ^2^ Although lymph node micrometastases sometimes cannot be detected by clinical examinations such as palpation, ultrasound, computed tomography (CT), and magnetic resonance imaging (MRI), they can be revealed by postoperative pathological examination. Such micrometastases are called occult lymph node metastases.^3^, ^4^ Up to 33% of OTSCC cases may have occult lymph node metastasis that is currently undetectable by imaging techniques.^5^ In patients with OTSCC, the survival rate of patients with cervical lymph node metastasis is 50% lower than that of patients without cervical lymph node metastasis.^6^ Therefore, preoperative diagnosis of cervical lymph node metastasis in patients with OTSCC is difficult but important in clinical practice. In addition, patients without lymph node metastasis (N_0_) tend to suffer unnecessary complications such as intraoperative risk and shoulder joint dysfunction if they receive cervical lymph node dissection, whereas if patients with occult lymph node metastases do not undergo lymph node dissection, they tend to have a high risk of short‐term local recurrence and poor prognosis.^7^

The early diagnosis of OTSCC and risk assessment of lymph node metastasis still lack readily available and reliable molecular markers. In the past few years, the relationship between tumors and inflammation has become a research hotspot. Tumor cells promote systemic inflammatory responses and cause changes in white blood cell (WBC) and platelet (PLT) counts in peripheral blood.^8^ They mainly mediate these changes by releasing stress‐related substances and proinflammatory cytokines, such as tumor necrosis factor α, which in turn can affect the occurrence and progression of tumors.^9^ Therefore, the neutrophil‐to‐lymphocyte ratio (NLR), the platelet‐to‐lymphocyte ratio (PLR), and the platelet‐to‐neutrophil ratio (PNR) of the peripheral blood obtained from a complete blood count have been used as indicators of systemic inflammation, and the diagnostic and prognostic value of NLR, PLR, and PNR for multiple tumors has been investigated. Current studies have found that NLR, PLR, and PNR have important reference value for the early diagnosis of lung cancer, liver cancer, breast cancer, and colorectal cancer as well as the prediction of lymph node metastasis.^10^, ^11^, ^12^, ^13^ However, the relationships of NLR and PNR with the occurrence and development of OTSCC have not been reported.

This study aimed to evaluate the value of NLR and PNR in peripheral blood for the diagnosis of OTSCC and the prediction of cervical lymph node metastasis to provide a reference for the development of surgical plans for OTSCC patients.

## 2 MATERIALS AND METHODS

### 2.1 General information

A total of 73 patients with primary OTSCC who underwent surgery at the Department of Oral Head and Neck Oncology of the Ninth People's Hospital affiliated to Shanghai JiaoTong University School of Medicine between January 2017 and December 2019 were enrolled. OTSCC was histopathologically confirmed in all patients. The exclusion criteria were as follows: (a) patients with other malignancies, (b) patients who had received radiotherapy or chemotherapy before surgery, (c) patients who could not be followed up, and (d) patients with active inflammation. At the same time, the information about age, sex, smoking history, drinking history, surgical records, pathological examination results, and tumor‐node‐metastasis (TNM) stage (according to the TNM classification of the American Joint Committee on Cancer (AJCC) in 2017) was collected.^14^ The OTSCC patients were divided into groups N_0_ (no early‐stage lymph node metastasis) and N_1_ (early‐stage lymph node metastasis). Early‐stage refers to primary tumor in T_1_ stage (tumor ≤2 cm with depth of invasion DOI ≤ 5 mm) or T_2_ stage (tumor ≤2 cm, with DOI > 5 mm and ≤10 mm; or tumor >2 cm and ≤4 cm, with DOI ≤ 10 mm). During the same period, 87 healthy individuals were included in the control group (group HC). This study was approved by the Ethics Committee of the Ninth People's Hospital affiliated to Shanghai JiaoTong University School of Medicine, and all participants signed informed consent forms.

### 2.2 Complete blood count

Venous blood samples of each OTSCC patient were collected before surgery and 3 weeks after the surgery. Each sample was immediately mixed with K2‐ethylenediaminetetraacetic acid (EDTA) in anticoagulation vacuum tubes. The sample was submitted to a complete blood count within 4 h of sample collection in a blood analyzer. Venous blood samples of patients in group HC were also collected for a complete blood count.

### 2.3 Statistical analysis

SPSS was used for statistical analysis. The independent‐samples *t* test, the chi‐squared test, and receiver operating characteristic (ROC) curve analysis were used for intergroup comparisons. *p* < 0.05 was considered statistically significant.

## 3 RESULTS

### 3.1 General information of patients

There was no significant difference in the general information between group N_0_ and group N_1_. The detailed results are shown in Table 1.

**Table 1:** General clinical information of group N_0_ and group N_1_

Variable	HC group	OTSCC group	*p*
	(*n* = 87)	(*n* = 73)	
Age	50.9 ± 9.4	53.7 ± 17.3	0.303
Gender			
Male	50	40	0.734
Female	37	33	
Smoker			
Yes	14	20	0.082
No	73	53	
Drinker			
Yes	5	7	0.358
No	82	66	

Note

Data are presented as numbers.

### 3.2 NLR level in OTSCC patients

**Figure 1:**
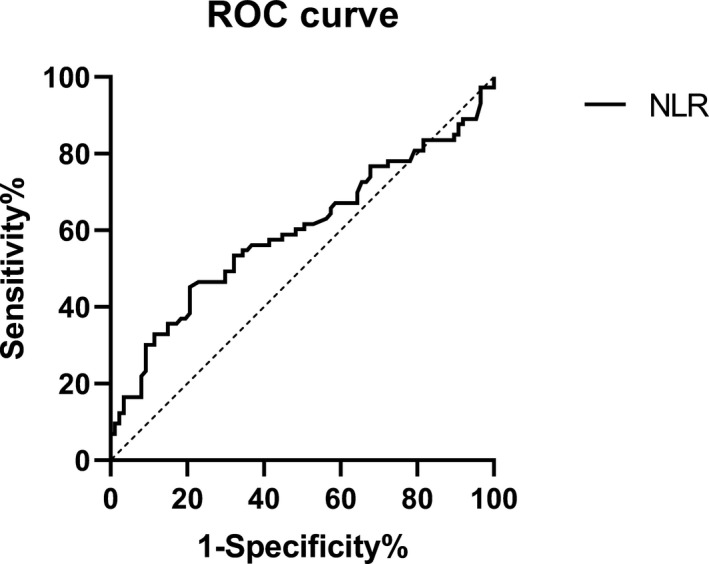
ROC curves of the NLR for the OTSCC patients and the controls

The OTSCC patients had lower lymphocyte and PLT counts than the controls (*p* < 0.05), though their neutrophil counts were similar (*p* > 0.05). The PLR and PNR of OTSCC patients were not significantly different from those of the controls, while the NLR level of OTSCC patients was higher than that of the controls (*p* < 0.05) (Table 2). The ROC curve analysis showed that the area under the ROC curve (AUC) of NLR was 0.595, with the 95% confidence interval (CI) of 0.504–0.686. The Youden index analysis showed that the threshold or cutoff value of the NLR was 2.059 and that the sensitivity and specificity of NLR to diagnose OTSCC were 46.6% and 79.3%, respectively (Figure 1).

**Table 2:** Complete blood count parameters and calculated values of PLR, NLR, and PNR for groups N_0_, N_1_, and HC

Groups	*N*	PLT (×10^9^/L)	LYMPH (×10^9^/L)	NEUT (×10^9^/L)	PLR	NLR	PNR
HC	87	226.44 ± 47.97	1.98 ± 0.42	3.30 ± 0.85	117.12 ± 30.32	1.72 ± 0.60	73.34 ± 22.07
Pretreatment							
N_0_	43	210.23 ± 60.69	1.78 ± 0.60^†^	3.85 ± 1.29^‡^, ^¶^	129.59 ± 48.66	2.33 ± 0.80^‡^, ^¶^	58.69 ± 21.79^‡^, ^¶^
N_1_	30	206.90 ± 67.79	1.84 ± 0.59	2.84 ± 1.71^§^	122.31 ± 57.20	1.62 ± 1.00^§^	87.27 ± 38.26^†^, ^§^
N_0_ + N_1_	73	208.86 ± 63.26^†^	1.80 ± 0.60^†^	3.44 ± 1.55	126.60 ± 52.08	2.03 ± 0.95^†^	70.43 ± 32.66
Three month after treatment							
N_0_	25	229.68 ± 152.98	1.50 ± 1.38	8.08 ± 4.01	193.88 ± 134.64	6.73 ± 4.52	33.70 ± 20.0
N_1_	26	217.23 ± 69.69	1.2 ± 0.41	6.63 ± 3.24	202.65 ± 98.62	6.18 ± 4.23	40.77 ± 20.43

Note

Data are presented as numbers, and the data were calculated by different etiologies.

Abbreviations: NLR, neutrophil‐to‐lymphocyte ratio; PLR, platelet‐to‐lymphocyte ratio; PNR, platelet‐to‐neutrophil ratio.

^†^ Compared with group HC, *p* < 0.05.

^‡^ Compared with group HC, *p* < 0.01.

^§^ Compared with group N_0_, *p* < 0.01.

^¶^ Compared with group N_1_, *p* < 0.01.

### 3.3 Evaluation of the predictive value of NLR and PNR for early‐stage lymph node metastasis in patients with histopathologically confirmed OTSCC

**Figure 2:**
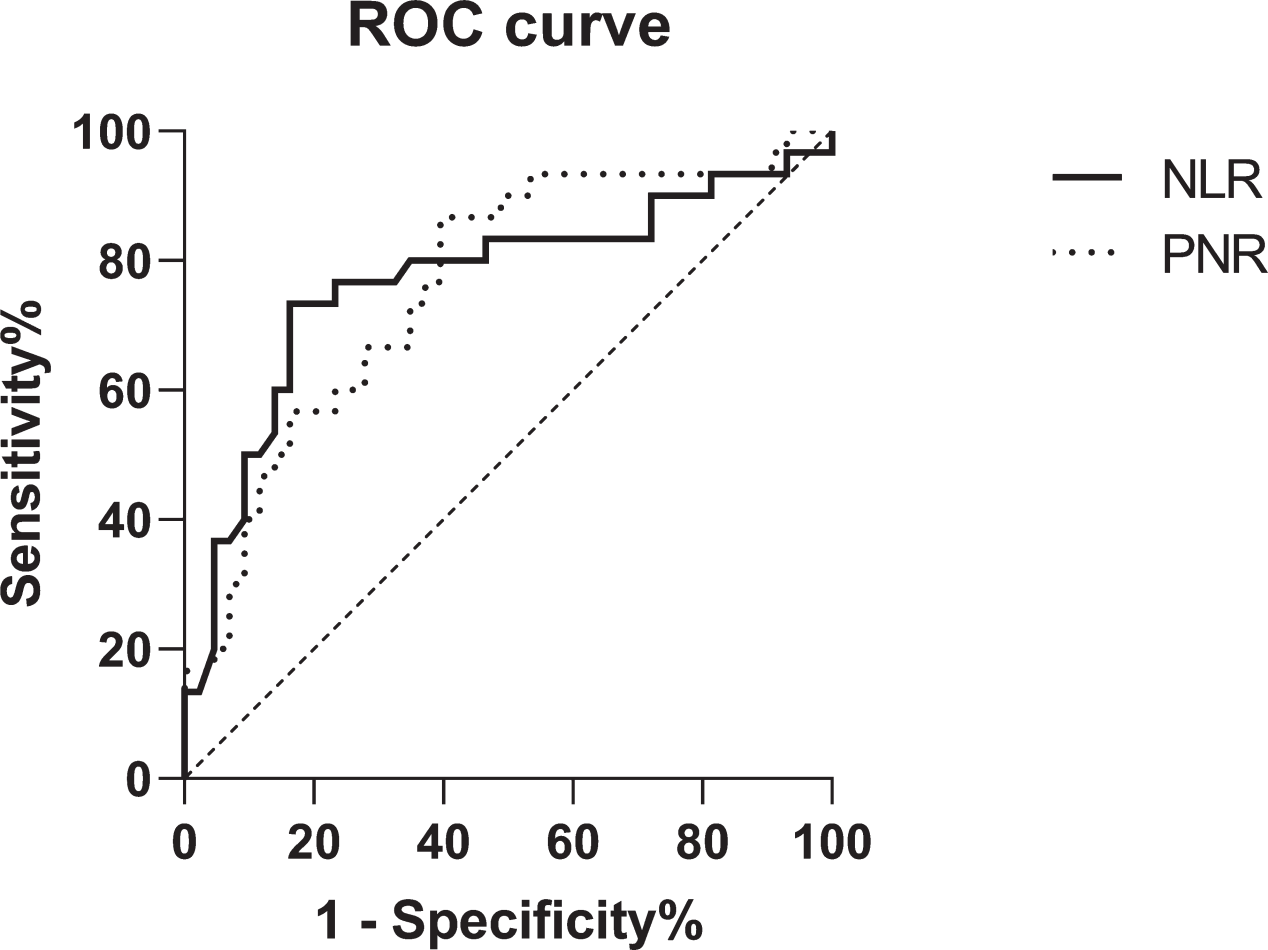
ROC curves of the NLR and PNR for groups N_0_ and N_1_

Compared with group HC, group N_0_ had a higher NLR (*p* = 0.0001) and lower PNR (*p* = 0.0001), and group N_1_ had a higher PNR (*p* = 0.017). Compared with group N_0_, group N_1_ had a lower NLR (*p* = 0.001) and higher PNR (*p* = 0.0001) (Table 2). Changes in NLR and PNR in groups N_0_ and N_1_ were analyzed using ROC curves. The NLR had the AUC of 0.769, the 95% CI of 0.649–0.889, the cutoff value of 1.6223, and the sensitivity of 73.3% in predicting early‐stage lymph node metastasis. The PNR had the AUC of 0.762, the 95% CI of 0.649–0.875, the cutoff value of 60.8892, and the sensitivity of 86.7% in predicting early‐stage lymph node metastasis (Figure 2, Table 3). However, at the third postoperative week, there was no significant difference in the NLR or PNR between groups N_0_ and N_1_ (Table 2).

**Table 3:** Assessment of early‐stage lymph node metastasis in OTSCC patients using the NLR and PNR

Samples	No. of cases	NLR		PNR	
		No. of positive	Positive rate (%)	No. of positive	Positive rate (%)
HC group	87	43	49.4	30	34.5
OTSCC group	73	29	39.7	43	58.9
N_0_ group	43	7	16.3	17	39.5
N_1_ group	30	22	73.3	26	86.7

Note

Data are presented as numbers and percentages, and the data were calculated by different etiologies.

## 4 DISCUSSION

There is a controversy regarding the treatment of cervical lymph nodes in N_0_ patients at the early stage of oral squamous cell carcinoma (OSCC). Some researchers advocate a wait‐and‐see strategy, which suggests that lymph node dissection should be performed only after confirmation of lymph node metastasis. However, this treatment regimen significantly reduces the survival rate of patients. Extended resection of the primary tumor(s) combined with cervical lymph node dissection has a high cure rate. Due to the extended scope of surgery, this may lead to early functional impairment or loss and thus affect the quality of life of the patients.^15^ Therefore, how to effectively assess noninvasive treatment for cervical lymph node–negative patients with enlarged cervical lymph nodes or a high risk for metastasis has been the focus of clinical studies.

This study first analyzed the NLR levels in the OTSCC group. Compared with group HC, the OTSCC group had a significantly higher NLR, mainly due to the lower absolute lymphocyte count and the (nonsignificantly) higher average absolute neutrophil count in the OTSCC group. According to the absence or presence of lymph node metastasis, the OTSCC group was divided into groups N_0_ and N_1_. This analysis showed that the absolute neutrophil count in group N_1_ was significantly lower than that in group N_0_ and that the absolute lymphocyte count and the absolute PLT count in group N_1_ were not significantly different from those in group N_0_. As a result, compared with group N_0_, group N_1_ had a significantly lower NLR, a significantly higher PNR, and a nonsignificantly different PLR. The causes for the differences between groups N_0_ and N_1_ might be that when the OTSCC did not metastasize (group N_0_), the innate immunity was dominant, so the neutrophil count was elevated. Our results did show that the absolute neutrophil count in group N_0_ was significantly higher than that in group HC.^16^ After tumor metastasis (group N_1_), the innate immunity was weakened, adaptive immunity began to play a dominant role, the neutrophil count dropped significantly lower than that before tumor metastasis (group N_0_), and the mean lymphocyte count was (nonsignificantly) larger than that in group N_0_, resulting in the lower NLR and the higher PNR in group N_1_.^17^, ^18^

Further analysis of groups N_0_ and N_1_ showed that the NLR and PNR had a sensitivity of 73.3% and 86.7%, respectively, in predicting early‐stage lymph node metastasis in patients with histopathologically confirmed OTSCC. Among OTSCC patients without lymph node metastasis, only 16.3% and 39.5% had “positive” NLR and PNR values, respectively. Therefore, NLR and PNR have good predictive value for early‐stage lymph node metastasis in OTSCC patients.

No previous studies have reported a relationship between lymph node metastasis in tongue cancer and the PNR. Xian et al^19^ studied 174 patients with solid carcinoma‐related cerebral venous sinus thrombosis and found that the higher PNR, the higher the probability of intracranial metastasis in these patients. This finding is similar to our finding that when PNR was >60.889, OTSCC patients were more prone to early‐stage lymph node metastasis, their innate immunity was weakened, their neutrophil count was decreased, and their PLT count was unchanged, resulting in an elevated PNR. Wu et al^20^ found that a high NLR (≥2.95) was significantly correlated with occult lymph node metastasis in OSCC. That result is inconsistent with ours. The possible cause of the discrepancy is that although OTSCC is an OSCC, it has its own characteristics, as a special OSCC with unique characteristics. This suggests that future studies on OSCC should refine their results by subtype and that the general conclusions drawn about OSCC are not necessarily applicable to all subtypes.

The NLR and PNR can be easily determined through a standardized, inexpensive process. One limitation of this study is that other systemic diseases may affect the NLR and PNR because they reflect the inflammatory state of the body. Second, this study was a retrospective single‐center study, so future multicenter studies are needed to verify our results. Third, the absolute counts of circulating neutrophils and lymphocytes were nonspecific, so there was no detailed information about the relationships of WBC subsets or activation status with OTSCC, such as whether a low activity of neutrophils or a high CD4/CD8 ratio is related to the progression of OTSCC. Nevertheless, our results strongly suggest that the preoperative NLR and PNR can be used as indicators to predict early‐stage lymph node metastasis in patients with histopathologically confirmed OTSCC.

## CONFLICT OF INTEREST

The authors declare that they have no competing interests.

## Data Availability

The raw/processed data required to reproduce these findings cannot be shared at this time as the data also forms part of an ongoing study.
